# Properties of Alkali-Activated Slag Paste Using New Colloidal Nano-Silica Mixing Method

**DOI:** 10.3390/ma12091571

**Published:** 2019-05-13

**Authors:** Taewan Kim, Jae Hong Kim, Yubin Jun

**Affiliations:** 1 Department of Civil Engineering, Pusan National University, Busan 46241, Korea; ring2014@naver.com; 2 Department of Civil and Environmental Engineering, Korea Advanced Institute of Science and Technology, Daejeon 34141, Korea; jae.kim@kaist.ac.kr

**Keywords:** alkali-activated slag cement, colloidal nano-silica, mixing-water, mixing method

## Abstract

Previous studies of alkali-activated slag cement (AASC) using nano-silica have mentioned mostly powdered nano-silica and binder weight replacement methods, which have a rapid decrease in fluidity, a short setting time and a low nano-silica replacement rate (< 5%). In this study, colloidal nano-silica (CNS) was used and the mixing-water weight substitution method was applied. The substitution method was newly applied to improve the dispersibility of nano-silica and to increase the substitution rate. In the experiment, the CNS was replaced by 0, 10, 20, 30, 40, and 50% of the mixing-water weight. As a result, as the substitution rate of CNS increased, the fluidity decreased, and the setting time decreased. High compressive strength values and increased rates were also observed, and the diameter and volume of pores decreased rapidly. In particular, the increase of CNS replacement rate had the greatest effect on decrease of medium capillary pores (50–10 nm) and increase of gel pores (< 10 nm). The new displacement method was able to replace up to 50% of the mixing water. As shown in the experimental results, despite the high substitution rate of 50%, the minimum fluidity of the mixture was secured, and a high-strength and compact matrix could be formed.

## 1. Introduction

Recently, many studies on the effect of nano-particles on the properties of cement and concrete have been reported [1,2]. Particularly, studies using various kinds of nano-particles centering on ordinary Portland cement (OPC) have been published [3,4,5]. So far, studies using nano-particles in the OPC base have shown improved mechanical properties and durability [6,7,8]. The cause of this improvement in OPC was found to be the nucleation effect, pozzolanic reaction and filler effect of nano-particles [9,10,11]. However, the research results of nano-particles based on OPC have pointed to a drastic decrease in fluidity as a major disadvantage [12,13]. The reason for the abrupt decrease in fluidity is the lack of mixing-water due to the surface area of high nano-particles [14,15]. This sudden drop in fluidity limits the use of nano-particles and limits the scope of nano-particle and OPC research. Previous researches have either increased the use of superplasticizers or added mixing-water to resolve the sudden drop in fluidity [16,17,18]. However, these methods have not been able to increase the use of nano-particles or to solve the problem of fluidity reduction fundamentally.

Although studies using nano-particles have been extended to alkali-activated cement (AAC) [16,17,18] and geopolymers [19,20,21,22,23,24,25], the study of nano-particles for AAC and geopolymers is relatively sparse compared to OPC [26,27]. In particular, the pozzolanic reaction of nano-particles mentioned in OPC is accompanied by the consumption of portlandite [15,28,29]. However, portlandite is not produced in AAC or geopolymer [30], which shows different effects and characteristics from OPC. In addition, AAC using alkali activation shows faster setting and hardening speeds than OPC. Therefore, AAC using nano-particles would require effective countermeasures to prevent fluidity reduction. Similar to OPC, studies on the high substitution rates of nano-particles in AAC are still insufficient. This is related to the problem of reduced fluidity with increasing substitution rate of nano-particles. In particular, the characterization of nano-silica and AAC/geopolymer by mixing method is rare [31].

In AAC or geopolymer studies using nano-silica particles, some researchers conducted studies on flowability and mixing properties using colloidal nano-silica instead of powdered nano-silica. Previous studies have reported on the effect of various additives, such as content of nano-silica [32,33], kind of activator [34], binder type [22,25,35,36,37], solid content rate of nano-silica [38], composition ratio of binder [19,26], concentration of activator [36], average diameter of nano-silica [37], and mixing method of nano-silica [31]. Most researchers mentioned a decrease in fluidity or a reduction in setting time as the content of nano-silica particles increases [21,27,34,35]. Also, the improvement of mechanical properties, such as increase in compressive strength and decrease of pores, have been mentioned [20,22,27,39,40,41,42,43]. These results are similar to those reported in OPC using nano-silica. It is known that colloidal nano-silica has better dispersibility of nano-silica particles compared with powdered nano-silica, and has superior initial strength improvement due to abrupt initial hydration reaction [30,44]. However, most of the nano-silicas used in OPC and AAC/geopolymer to date are of the powder type, and relatively few are of the colloidal type. 

The purpose of this study is to investigate the properties of the new nano-silica substitution method in AAC based on slag experimentally. For this, liquid colloidal nano-silica (CNS) was used instead of conventional powdered nano-silica. In addition, the mixing-water weight substitution method, which is not a binder weight substitution or addition method applied in previous studies on OPC, AAC and geopolymer, was applied. The effect of the new mixing method on the characteristics of alkali-activated slag cement (AASC) is investigated.

## 2. Materials and Methods

### 2.1. Materials

**Figure 1:**
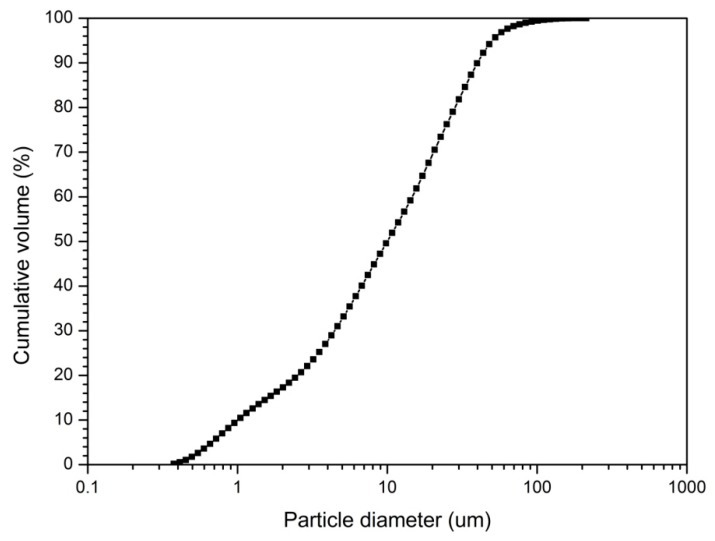
Particle analysis of slag.

Ground granulated blast-furnace slag (slag) was used as a single binder and its chemical constituents are shown in Table 1 from X-ray fluorescence analysis (XRF, SHIMADZU XRF-1800, Tokyo, Japan). The particle size distribution of slag is shown in Figure 1. Slag particle size analysis was performed using a Laser Diffraction Particle Size Analyzer (LS I3 320, Beckman Coulter, Inianapolis, USA). From the particle size graph, the median diameter (d_50_) of the slag particles is 11.15 μm and the mean diameter (d_mean_) is 18.75 μm. Sodium hydroxide (NaOH) and sodium silicate (Na_2_SiO_3_). were used as the alkaline activators. Commercial sodium silicate solution (liquid type, Ms = 2.0) and analytical grade sodium hydroxide pellets (purity: ≥98.0%) were used. The mixing ratio was 1:1 by weight of NaOH and Na_2_SiO_3_.

The activator concentrations were 5 and 10% of binder weight, where 5% activator means 5% NaOH + 5% Na_2_SiO_3_ and 10% activator means 10% NaOH + 10% Na_2_SiO_3_. No additional superplasticizer was used. A commercial-grade colloidal nano-silica (CNS) was used, which exhibited an average primary particle size of 20 nm, a density of 1.2 g/cm^3^, and an aqueous solution having an alkaline pH of 10. The SiO_2_ content in the aqueous solution was 30%, while the viscosity was reported to be not greater than 20 mPa·s at 20 °C.

### 2.2. Experimental Details

This study applied a mixing-water weight substitution method different from the binder weight substitution method used in previous studies. Activator solution was prepared by adding activator to mixing-water, slowly stirring, and left at room temperature for 3 hours. Then add CNS to the activator solution before mixing with the binder and stir well.

The water-to-binder ratio (w/b) of all paste mixtures was 0.5. Superplasticizer was not used in the present study. Therefore, w/b = 0.5 was selected as the range of mixing without the use of superplasticizer. The w/b was determined through preliminary experiments to find a mixture suitable for the purpose of this study using a CNS without using a superplasticizer. CNS replaced 0, 10, 20, 30, 40, and 50% of the mixing-water weight.

Table 2 shows the ratio of CNS to mixing-water weight displacement method (“CNS to mixing water (%)”). Because CNS is more dense than mixing-water, mixing-water decreases as the substitution rate of CNS increases. This leads to the effect of decreasing the w/b ratio. The reduction of w/b ratio due to substitution of CNS was calculated (“w/b considering water including CNS”). The ratio of CNS to binder weight (“CNS to binder ratio (%)”) and the ratio of SiO_2_ content in CNS were also calculated for binder weight (“Ratio of SiO_2_ in CNS to binder weight (%)”). The experiment was designed with 6 mixtures according to the CNS replacement rate shown in Table 2. Fifteen samples were prepared for one substitution rate (for each mixture).

Flow values was measured by using the instruments and procedure specified in ASTM C230 [45]. The setting time of alkali-activated slag cement (AASC) paste was measured using the instruments and procedure specified in ASTM C266 [46]. The paste was mixing procedure and the mixing time was measured according to the method specified in ASTM C305 [47]. After mixing, the paste was kept in a constant temperature and humidity chamber at a relative humidity (RH) of 90 ± 5% and temperature of 23 ± 2 °C for 24 hours. The mold was removed, and samples were stored in a chamber at RH ± 5%, 23 ± 2 °C until measurement date.

The compressive strength of paste samples was measured according to ASTM C109 [48] by using a 50 mm × 50 mm × 50 mm cube mold. The compressive strength of the paste was measured at 1, 3, 7, and 28 days. The compressive strength of three samples at each measurement day was measured and the mean value was used.

This powder sample was subjected to X-ray diffractometer (XRD, PANalytical Empyrean, Almelo, Netherlands) analysis. After measuring the compressive strength, the broken sample pieces were immersed in acetone for 12 hours and then dried in a vacuum desiccator for 24 hours. The dried sample was pulverized and subjected to XRD measurement. The XRD pattern was measured in the range of 5° to 60° in 2θ, and the step size was 0.017° (2θ) at 40 kV, 40 mA.

Pore structure analysis was done by mercury intrusion porosimetry (MIP, micromeritics AutoPore IV9500, Georgia, USA). The sample for MIP measurement was cut into a piece of cube of about 5 mm. The sample pieces were immersed in acetone for 12 hours, then dried in a vacuum desiccator for 24 hours and then measured. The conditions applied for the measurements are pore diameters of 0.003‒336 μm, contact angle of 130°, mercury density of 13.534 g/mL and surface tension of 485 mN/m. Scanning electron microscopy (SEM, Zeiss SUPRA ™ 40, Oberkochen, Germany) was performed on the 28-day sample to identify hydration reactants. Also, Energy Dispersive X-ray Spectrometry (EDS, Elite, AMETEK, New Jersey, USA) analysis was performed on hydration reactants. The measurement was carried out under the condition of accelerating voltage 15 kV in the high vacuum mode. For the SEM measurement, the sample was subjected to hydration stop treatment. For this purpose, the central part of the 28-day sample was cut into small pieces and then immersed in acetone for 12 hours and then dried in a vacuum desiccator for 24 hours. The dried sample is mounted with epoxy and stored in a vacuum desiccator for 24 hours. Samples were polished with SiC grinding disks of No.1200 and No.2000 and then polished with 9, 6 and 3 μm diamond suspensions. Platinum coating was applied to the polished surface before SEM observation. Moreover, the samples were subjected to thermogravimetric (TG) and differential thermo gravimetry (DTG) analyses at 28 days of age. A DSC800 (Perkin Elmer, Massachusetts, USA) thermal analyzer was used, and the samples were measured at temperatures of 30 °C to 800 °C at a heating rate of 20 °C /min in N_2_ gas environment.

## 3. Results and Discussion

### 3.1. Flow and Setting Time

**Figure 2:**
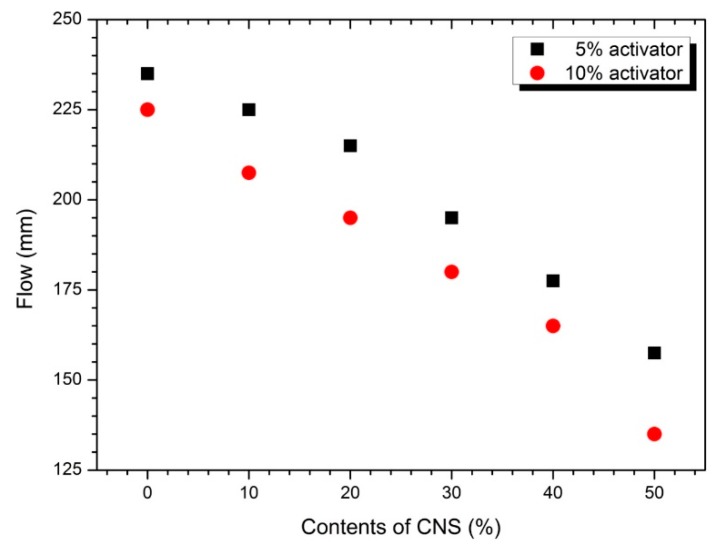
Flow values according to CNS replacement ratios in 5% and 10% activators.

Figure 2 shows the flow values for various CNS replacement rates for AASC paste with 5% and 10% activators. Regardless of the activator concentration, the flow value of the paste decreased with increasing CNS replacement ratio. The activation of slag increases when the concentration of activator increases from 5% to 10%. As a result, the hydration reaction rate increases and the amount of hydration reactant increases, so the viscosity of the paste increases and the flow value decreases. The mixing-water weight substitution method applied in this experiment decreases the w/b ratio as the substitution rate of CNS increases. Therefore, the concentration effect of the activator and the w/b reduction effect on the CNS substitution influence the decrease of the flow value.

Gao et al. [19] performed a paste experiment in which nano-silica slurry (solid content of 50 wt.% and d_50_ of 0.12 μm) was replaced with 0, 1, 2, and 3% of the binder weight in the binders having GGBS/FA composition ratios of 70/30 and 30/70. Experimental results show that the slump flow decreases steeply as the nano-silica contents increase from 0 to 3%. This fluidity reduction has been explained by the high reactivity of nano-silica particles containing unsaturated Si-O bonds that a certain amount of water in solution can be bound around the nano-silica particles with formation of Si-OH [32,33]. Therefore, the presence of nano-silica reduces the amount of mixed water required for hydration of slag and fly ash, which contributes to the decrease in the fluidity of the paste. In addition, Behfarnia and Rostami [21] and Ramezanianpour and Moeini [34] have reported a similar tendency of decreasing flow as the amount of nano-silica increased.

However, some researchers have also reported different trends of flow or workability properties depending on the amount of nano-silica.

Ibrahim et al. [35] reported different flow characteristics in the flow of alkali-activated mortar using colloidal nano-silica (50% solids content, average particle size 35 nm and pH 9.5) and natural pozzolan as a binder. They reported that the flow rates were 164, 170, 172, 158 and 152 mm as the replacement rates of colloidal nano-silica increased to 0, 1, 2.5, 5.0 and 7.5%, respectively. Flow results show a slight increase with increasing colloidal nano-silica up to 2.5%, but a decrease from 5.0%. This fluidity change was noted as a slight increase in flow due to colloidal nature in the range of as low as 2.5% of the replacement rate of colloidal nano-silica. Also, the flow reduction seen over the 5.0% colloidal nano-silica range was explained by the increased demand for nano-silica mixed water with greater specific surface area.

Adak et al. [36] was tested with colloidal nano-silica (30% solids content, pH 9.0–9.6, average particle size 4–16 μm) and a 12M activator concentration in mortar with fly ash:sand = 1:3 ratio. In the experiments, colloidal nano-silica was added at 4, 6, 8 and 10% of the fly ash weight. As a result, the slump gradually increased with increasing colloidal nano-silica. This is due to the difference in the method of adding colloidal nano-silica, not substitution.

Deb et al. [37] reported the experimental results of substituting 0, 1, 2 and 3% of powdered nano-silica for three types of binders, fly ash, ground granulated blast furnace slag (GGBFS)andordinary Portland cement (OPC). As a result, the flow value decreased as the content of nano-silica increased in all three types of binder. 

In geopolymer studies using various nano-particles, nano-particles seem to have a greater effect of reducing fluidity (or workability) and reducing setting time [27,38]. Therefore, the flow or workability of the nano-silica depends on the nature of the nano-silica, the amount used, the method of substitution or addition.

**Figure 3:**
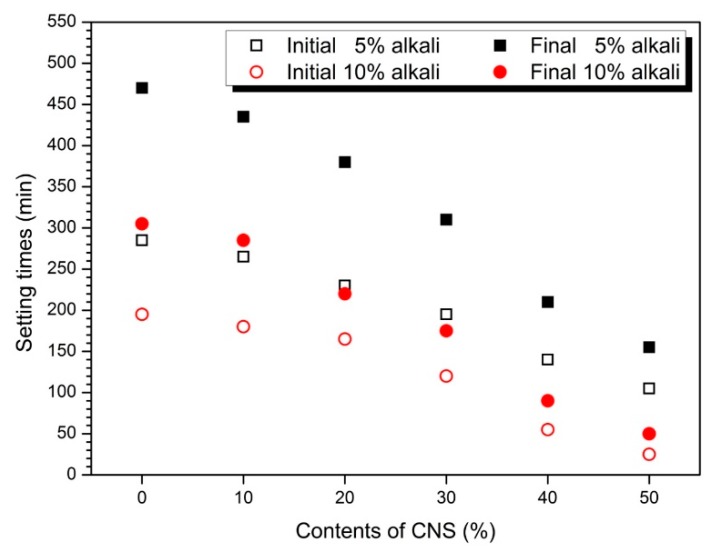
Setting times according to CNS replacement ratios in 5% and 10% activators.

Figure 3 shows the setting time measurement results. As the amount of CNS increased, both initial and final decreased. Also, the setting time of 10% activator samples was faster than 5%. As the substitution rate of the CNS increases, the setting time reduction in Figure 2 and the flow value decrease tendency in Figure 1 are similar.

Gao et al. [19] reported a gradual increase in setting time as nano-silica contents increased to 0, 1, 2 and 3% in alkali-activated slag-fly ash blends paste. Adak et al. [36] increased colloidal nano-silica by 4, 6, 8, and 10% in the fly ash-based geopolymer mortar experiment by adding colloidal nano-silica. As a result, it was reported that the setting time increases with increasing amount of colloidal nano-silica.

Ibrahim et al. [35] reported setting time results after 0, 1, 2.5, 5.0 and 7.5% substitution of colloidal nano-silica in natural pozzolan experiments activated with sodium silicate and sodium hydroxide. As a result, setting time decreased until colloidal nano-silica replacement rate to 5%. However, 7.5% colloidal nano-silica showed a slightly longer setting time than 5%. In Phoo-ngernkham et al. [39], an experiment was carried out to add 0, 1, 2 and 3% of powdered nano-silica to FA-based geopolymer. As a result, setting time decreased with increasing amount of nano-silica.

Setting time is influenced by various factors such as nano-silica nature (solid contents ratio, average size), type (powder or colloidal), mixing method (added or substitution), binder type (fly ash, slag, metakaoline), type and concentration of activator, respectively [27,38].

In previous AAC or geopolymer studies using nano-silica, curing time was shortened because nano-silica promotes the production and growth of reaction products [19]. In addition, the mxing-water substitution method used in this study is an additional factor that reduces the setting time by the w/b reduction effect with increasing CNS substitution rate.

### 3.2. Compressive Strength

**Figure 4:**
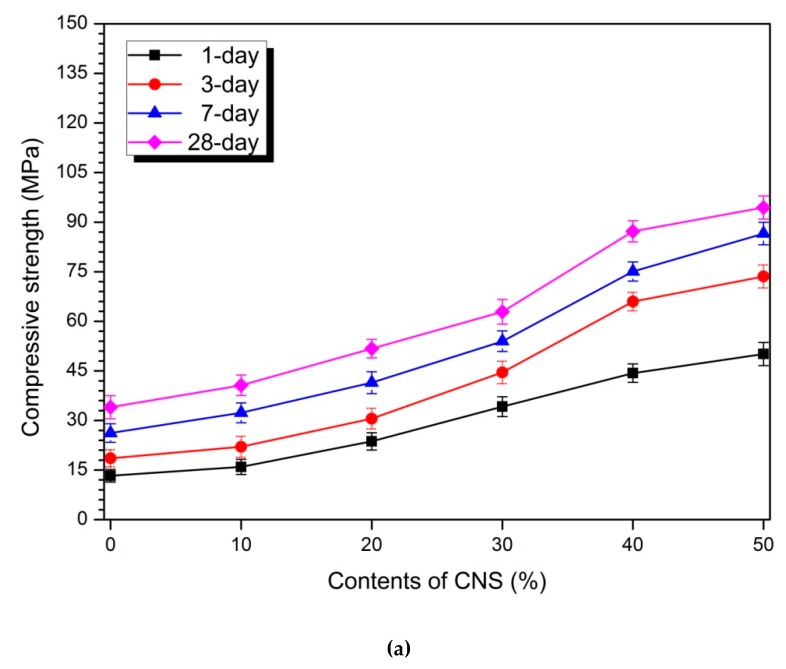

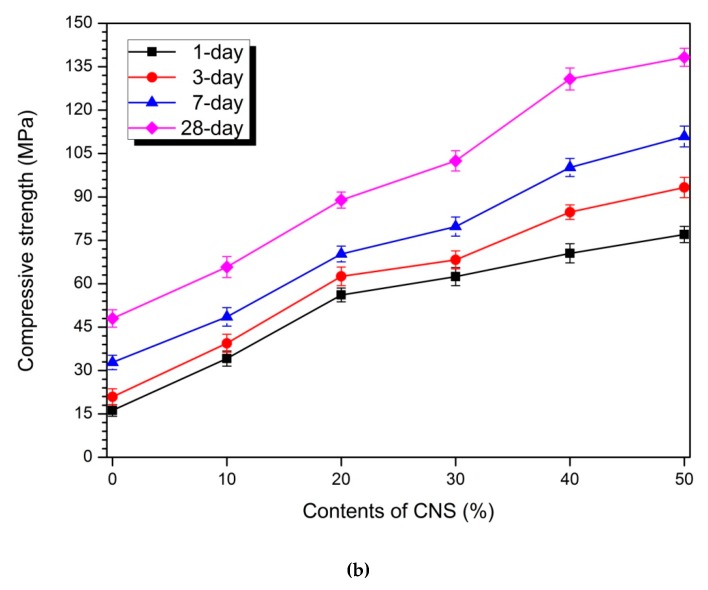
Compressive strength: (**a**) 5% activator and (**b**) 10% activator samples.

Figure 4 shows the compressive strength values of the samples with 5 and 10% activator. The compressive strength of the samples containing 5 and 10% activator increased at each measurement age as the CNS replacement ratio increased. As the age increased, the compressive strength increased. Figure 4a shows the measured strength values of the samples with 5% activator. The 1-day strength values of the 0% CNS and 50% CNS samples were 13.3 MPa and 50.1 MPa, respectively. The 3-, 7-, and 28-day strengths of the 0% CNS and 50% CNS samples were 18.6 MPa, 26.2 MPa, and 34.0 MPa and 73.6 MPa, 86.5 MPa, and 94.4 MPa, respectively.

Strength improvement due to CNS substitution was observed at all measurement ages, and the measured compressive strength increased with increasing CNS replacement ratio. The compressive strength of the 10% activator samples for various CNS replacement ratios is shown in Figure 4b. The strengths of the samples containing 0 and 50% CNS were 16.2 MPa and 77.0 MPa at 1 day, 20.9 MPa and 93.3 MPa at 3 days, 32.8 and 110.8 MPa at 7 days, and 48.0 MPa and 138.24 MPa at 28 days, respectively. Similar to the trend shown by the 5% activator samples, the compressive strength of the 10% activator samples increased with increasing CNS replacement ratio. No unusual trends were found in the variation of compressive strength by measurement age with increasing CNS replacement ratio.

The enhancement of strength due to the use of nano-particle in Portland cement has already been revealed by many previous studies [6,7]. The effect of OPC-based nano-particles on the strength enhancement is known to be due to pozzolanic reaction, nucleation effect and filler effect [9,10].

Some researchers have also reported on the effects of nano-particles on the mechanical performance of AAC or geopolymer. Some studies have reported that there is an optimal nano-silica content with the highest strength. Gao et al. [22] performed an alkali-activated metakaolin experiment using powdered nano-silica. In the experiment, nano-silica added 0, 1, 2, and 3% of the weight of metakaolin as a binder. The flexural strength results show that 1% nano-silica is capable of high strength. The strength results of Gao et al. [22] suggest that the content of nano-silica with optimal strength is present. Gao et al. [19] studies showed strength results for nano-silica slurry and alkali-activated slag/fly ash cement. Gao et al. [19] replaced 0, 1, 2 and 3 of nano-silica with respect to the weight of slag/fly ash as a binder. As a result, 2% nano-silica sample showed the highest compressive strength at all measurement ages. Wang et al. [40] performed an experiment using powdered nano-silica with two kinds of average particle sizes (30 nm, 15 nm) in AASC. 30 nm nano-silica was 0.5 to 6.0%, and 15 nm nano-silica was 0.5 to 2.5%. As a result, the maximum compressive strength was measured at 3.0% for 30 nm nano-silica and at 2.0% for 15 nm nano-silica. Experimental results of Wang et al. [40] confirmed that the content of nano-silica, which gives the highest strength depending on the particle size of nano-silica, is affected. Ibrahim et al. [35] performed an experiment in which the colloidal nano-silica was replaced with 0, 1, 2.5, 5, and 7.5% of the binder weight in the alkali-activated natural pozzolan experiment. As a result, 5% colloidal nano-silica showed the highest compressive strength. Adak et al. [36] carried out an experiment in which colloidal nano-silica was added at 4%, 6%, 8% and 10% in 12M concentration activator and fly ash-based geopolymer. Experimental results indicated that the highest compressive strength was 6% colloidal nano-silica. In addition, Deb et al. [41] performed experiments using fly ash-only-based geopolymer, slag blended fly ash-based geopolymer and powdered nano-silica. Nano-silica was substituted for 0, 0.5, 1, 1.5, 2, 2.5, and 3% of binder weight. Fly ash-only-based geopolymer and slag-blended fly ash-based geopolymer showed the highest compressive strength in 2% nano-silica. Deb et al. [37] reported that fly ash-based geopolymer experiments with 0, 1, 2, and 3% substitution of powdered nano-silica were performed, and the maximum strength was measured on a 2% nano-silica sample. Behfarnia and Rostami [21] reported that the highest strength was obtained at 3% in AAC experiments where powdered nano-silica was replaced with 0, 0.5, 1, 3, and 5% of slag weight.

However, other researchers reported that as the content of nano-silica increases, the compressive strength increases. Shahrajabian and Behfarnia [42] reported that compressive strength increased with increasing amount of nano-silica in the AASC experiment using 0, 1, 2, and 3% of powdered nano-silica, and the maximum strength was 3%. In a study of Yang et al. [43] in which 0.5% of nano-TiO_2_ was mixed with alkali-activated slag pastes, the mixing of nano-TiO_2_ exhibited an increase in compressive strength and flexural strength. Long et al. [20] showed that the 2% replacement sample showed the highest compressive strength and flexural strength in an alkali-activated slag cement with 0 and 2% replacement of colloidal nano-silica. Prakasam et al. [26] produced a geopolymer of binder with slag:fly ash = 50:50 using powdered nano-silica at 0, 0.5, and 1.0%. strength test showed that 2.0% nano-silica had the highest compressive strength at all measurement times.

The filler effect and nucleation effect have been mentioned as the causes of the strength enhancement of nano-particles in AAC/geopolymer [19,22,23,24,35,40]. In AAC/geopolymer, the pozzolanic reaction was excluded from the strength enhancement effects of OPC-based nano-particles. This is because there is no portlandite required for pozzolanic reaction in the hydration reaction of AAC/geopolymer [30].

The strength characteristics of AAC or geopolymer using nano-silica are affected by various factors, and the variation of the strength improvement effect is also great [27,38]. However, the experimental factors and the mixing-water weight substitution method considered in this study tend to increase the compressive strength with increasing CNS replacement ratio. This is because the effect of increasing the strength of nano-silica already known in previous studies and the effect of w/b ratio reduction by mixing-water weight substitution method are also affected. It was also found that there was no decrease in compressive strength during replacement of the CNS up to 50%. This is because the mixing-water weight substitution method of the CNS improves the dispersibility by reducing the aggregation or aggregation of the nano-silica particles. Therefore, it was confirmed that the weight substitution method of mixing-water by CNS is an effective mixing method for strength improvement.

Table 3 calculates the compressive strength increase rate of CNS substituted samples for 0% CNS. Table 3 shows that the increase rate of compressive strength increases with the substitution rate of CNS regardless of the concentration of activator. In addition, the 1-day compressive strength increase rate of the measurement period was the largest increase rate compared with the increase rate in the remaining ages. This means that the strength enhancement effect of CNS appears to be highest within 1 day of the initial stage of hydration reaction.

In the study of OPC, the hydration reaction of nano-silica at the initial hydration stage and the effect of improving the strength at the early ages have already been reported. In particular, colloidal nano-silica has been reported to form C-S-H gel in the initial hydration stage before 12 hours [44]. The effect of accelerating the initial hydration of CNS in OPC is similar in alkali-activated slag paste using CNS considered in this study. As shown in Table 3, the highest compressive strength increase rate was 1-day. As the age increases, the rate of increase of the compressive strength according to the substitution rate of CNS gradually decreases. This indicates that the strength improvement effect of CNS is highest at the initial stage of hydration reaction and gradually decreases with time. Therefore, in order to improve the strength of CNS-substituted AASC, the initial hydration reaction step is important. To this end, the mixing method should be adjusted so that a sufficiently even dispersion of the nano-silica particles occurs during the initial hydration stage.

### 3.3. Hydration Products

**Figure 5:**
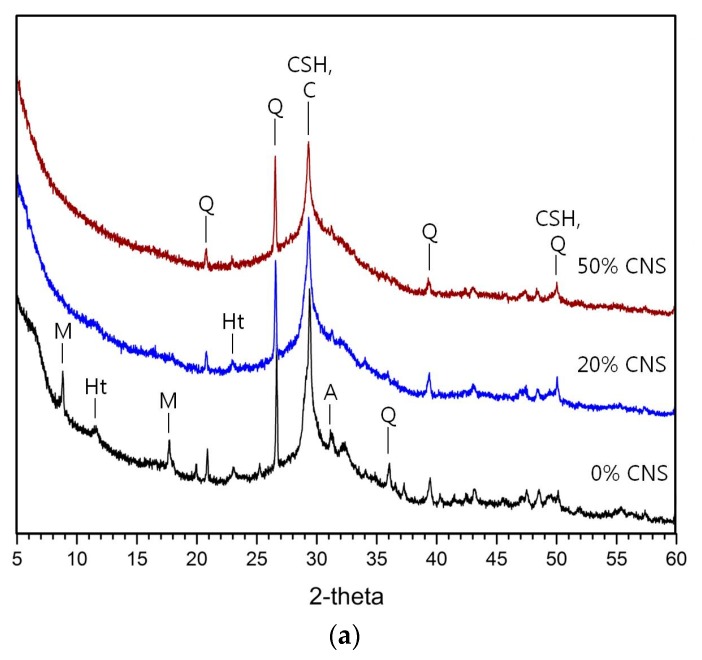

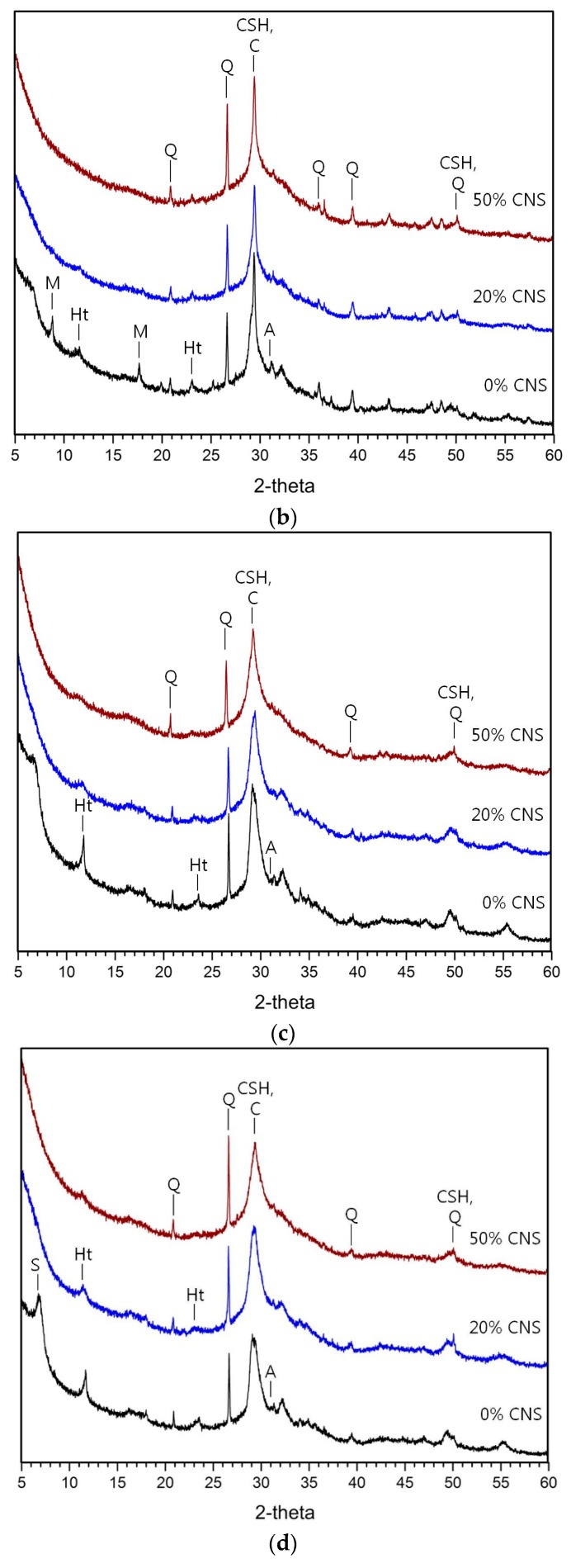
XRD analysis of hydration reactants: (**a**) 1-day, 5% activator samples (**b**) 28-day, 5% activator samples, (**c**) 1-day, 10% activator samples, (**d**) 28-day, 10% activator samples. M is monosulfate, C is calcite, CSH is C-S-H gel, Ht is hydrotalcite, Q is quartz, A is akermanite, S is stratlingite.

Figure 5 shows the XRD analysis of hydration reactants at 1-day and 28-day for 0, 20, and 50% CNS samples. The major reactants were C-S-H gel, calcite, hydratalcite, monosulfate and quartz. Regardless of the concentration of activator, akermanite, which was observed in 0% CNS, decreased with increasing CNS replacement rates of 20 and 50%. Monosulfate and hydrotalcite, which were observed in 0% CNS, also decreased gradually and were not observed in 50% CNS samples. However, C-S-H gels, known as the major hydration reactants of AASC, show complex and irregular changes without a consistent tendency as the substitution rate of the CNS increases. C-A-S-H gels play a central role in improving the mechanical properties and durability of AASC. Therefore, the use of CNS promoted the hydration reaction of slag and expected to increase the amount of C-S-H gel. However, the XRD analysis does not clearly show the tendency to change the C-S-H gel due to the substitution of the CNS. The C-S-H gels are amorphous and cause difficulty in understanding the exact trend because of the overlap of calcite and peak. 

Some researchers reported that nano-silica had little effect on the formation of C-S-H gel. Long et al. [20] mentioned similar trends of C-S-H gel peaks in XRD patterns of 0 and 2% nano-silica samples in AASC. It is reported that the mixing of nano-silica does not contribute to the formation of another polymerization product in AASC. Assaedi et al. [31] mentioned a small change in crystalline and amorphous contents in geopolymers mixed with 0.5, 1.0, 2.0, and 3.0 wt.% nano-silica.

As seen from the results of Long et al. [20] and Assaedi et al. [31], previous studies have not found a clear contribution to the change of C-S-H gel by nano-silica particles in AASC. This is because it is difficult to quantitatively evaluate the effect of nano-silica particles on nucleation and filler effects. Therefore, formation of hydration reactants such as C-S-H gel is insufficient to explain the cause of improvement of strength by nano-silica particles. 

From the results of XRD analysis in Figure 4, the effect of CNS on the formation of C-S-H gel is small or overlapping with the calcite peak, making it difficult to grasp the clear tendency. To evaluate the effect of CNS on the formation of C-S-H gel, it is concluded that XRD and other analyses should be performed in parallel.

### 3.4. Pore Structure

**Figure 6:**
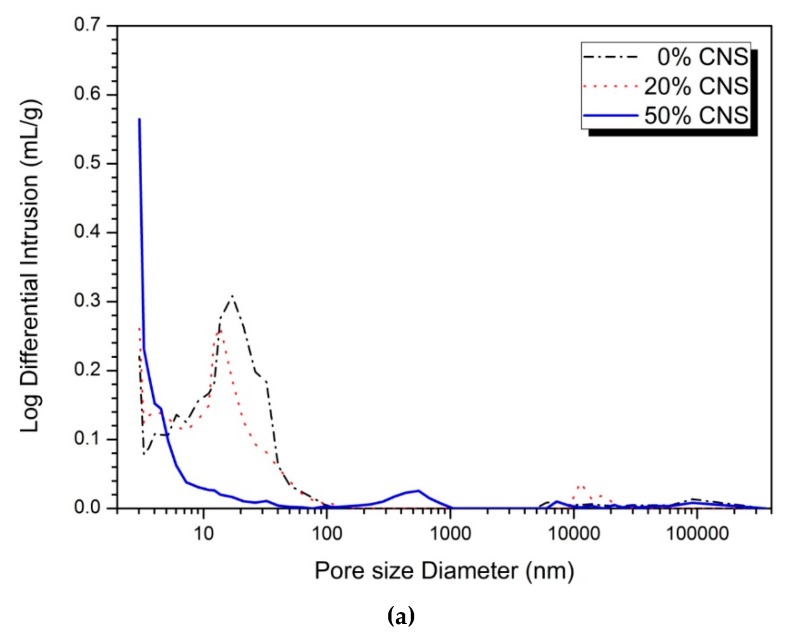

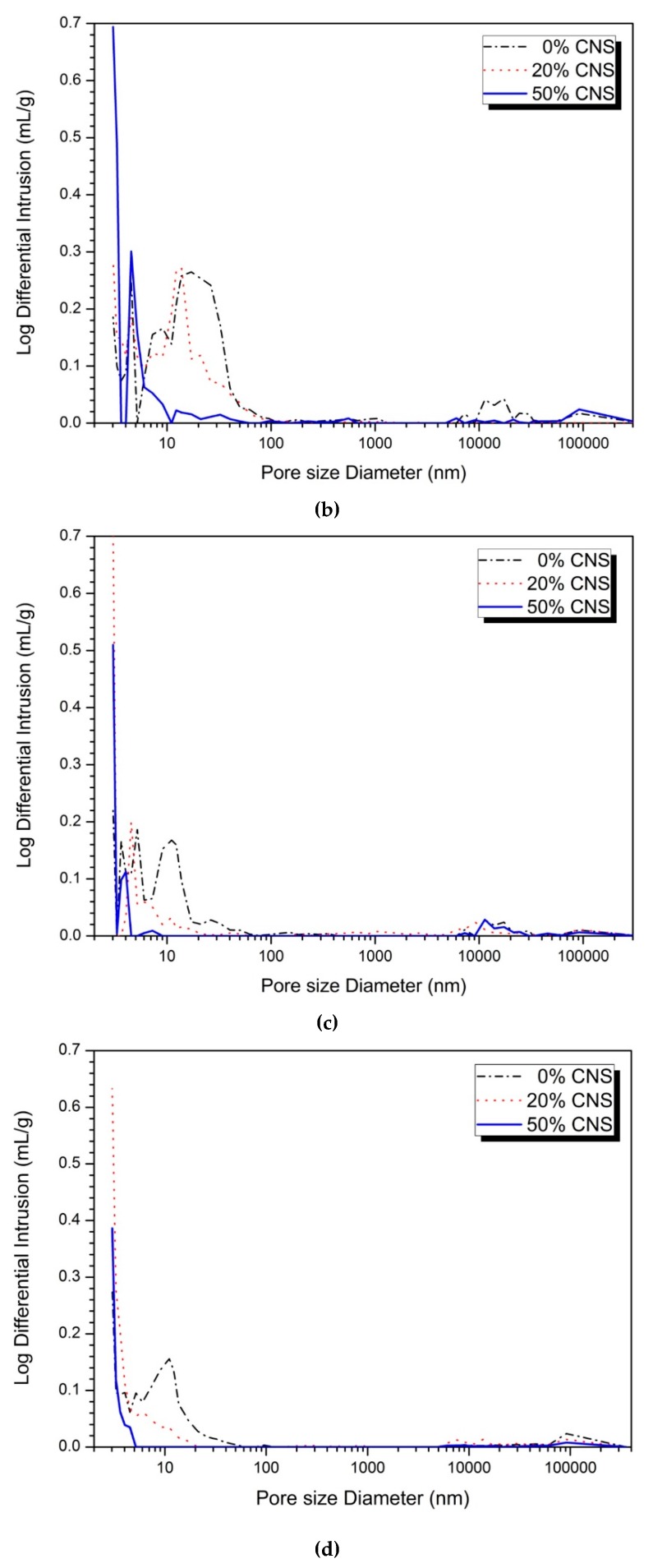
MIP analysis: (**a**) 5% activator, 1-day, (**b**) 5% activator, 28-day, (**c**) 10% activator, 1-day, (**d**) 10% activator, 28-day.

Figure 6 shows the MIP results for analyzing the void structure of the 0, 20, and 50% CNS samples of the 5 and 10% activator. Comparing the porosity distributions of the 5 and 10% activator samples, the 10% activator showed a decrease in pore size and volume. This is because, as the concentration of the activator increases, the hydration reaction of the slag is promoted and the amount of hydration reaction product is increased to form a dense matrix. Also, no significant change in pore diameter or volume was observed when comparing the pore distribution after 1 day and 28 days, regardless of the concentration of activator.

In the pore structure analysis of Gao et al. [22], 1% nano-silica sample showed the lowest porosity in the experiments in which 0, 1, 2, and 3% of powdered nano-silica were replaced. The highest compressive strength was also measured on a 1% nano-silica sample. Wang et al. [40], which reported the results of AASC experiments in which nano-silica was replaced by 2% and 3% of the weight of the binder, also showed a decrease in pore size and volume due to substitution of nano-silica. In a study of Yang et al. [43] for AAS paste with 0.5% nano-TiO_2_ substitution, TiO_2_ reduced the total porosity and the amount of pores larger than 25 nm. Long et al. [20] reported that pore size and volume decreased in AASC experiments using 0 and 2% colloidal nano-silica.

**Figure 7:**
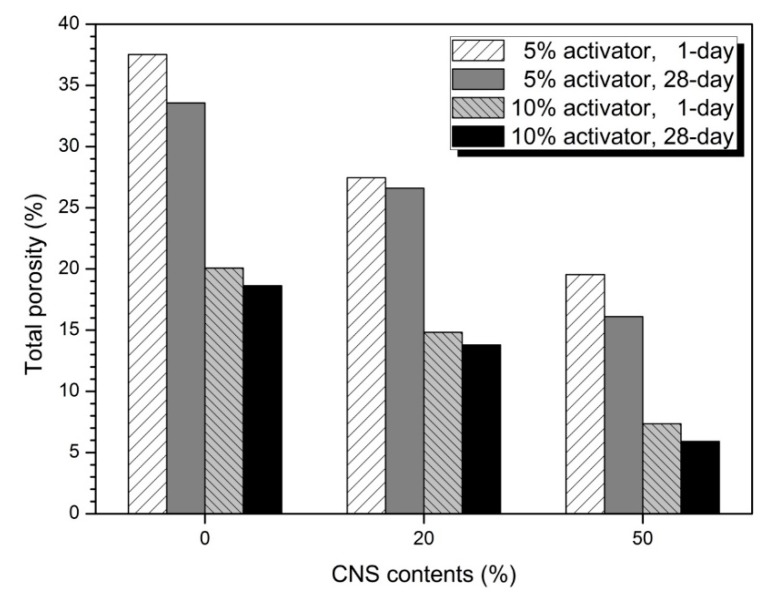
Total porosity of samples.

Figure 7 shows the total porosity of 0, 20, and 50% CNS. As the substitution rate of CNS increased, total porosity decreased. Total porosity at 5 and 10% activator samples was slightly lower than 1-day at 28-day. The total porosity of the 10% activator sample was also lower than that of the 5% activator. The difference in total porosity between 1-day and 28-day was small. This means that the effect of densification of the matrix by the CNS after 1-day becomes insignificant. This supports the result that the above-mentioned maximum compressive strength increase rate is 1-day.

Table 4 shows the composition ratio of pores of 10,000 nm or less [49]. The medium capillary pore gradually decreased and the gel pore increased as the CNS replacement ratio increased in the 5% activator sample. In the 28-day sample, the medium capillary pore decreased and the gel pore increased as compared to the 1-day. The 10% activator sample showed similar tendency to 5% activator. As the substitution rate and age of the CNS increase, the medium capillary pore decreases and the gel pore increases. It was also found that 10% activator samples had more gel pores than 5% activator. Therefore, the substitution of the CNS with increasing concentration of the activator decreases the size and amount of pores by making the hydration reaction matrix dense. This is due to nucleation and filler effects [24]. In Table 4, no significant difference in the ratio of pore sizes between 1-day and 28-day was observed, regardless of the concentration of activator. This means that the effect on the pore structure by the hydration reaction after 1-day is relatively small. Therefore, the compressive strength increase rate described in Table 3 was highest at 1-day and lowest at 28-day.

### 3.5. Thermal Analysis

**Figure 8:**
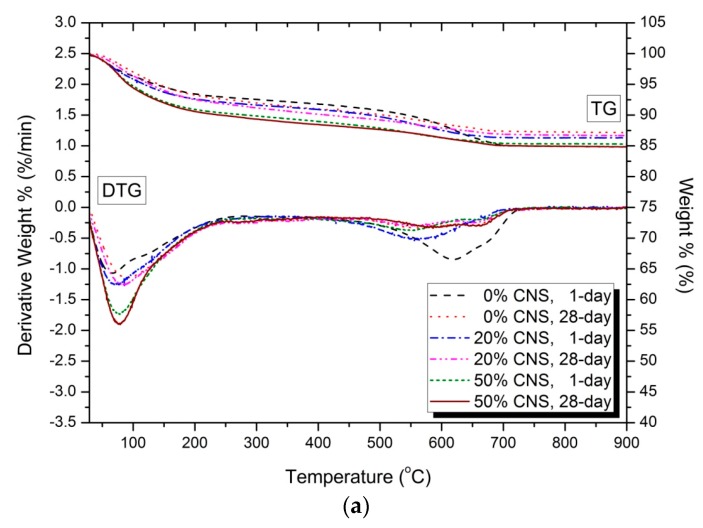

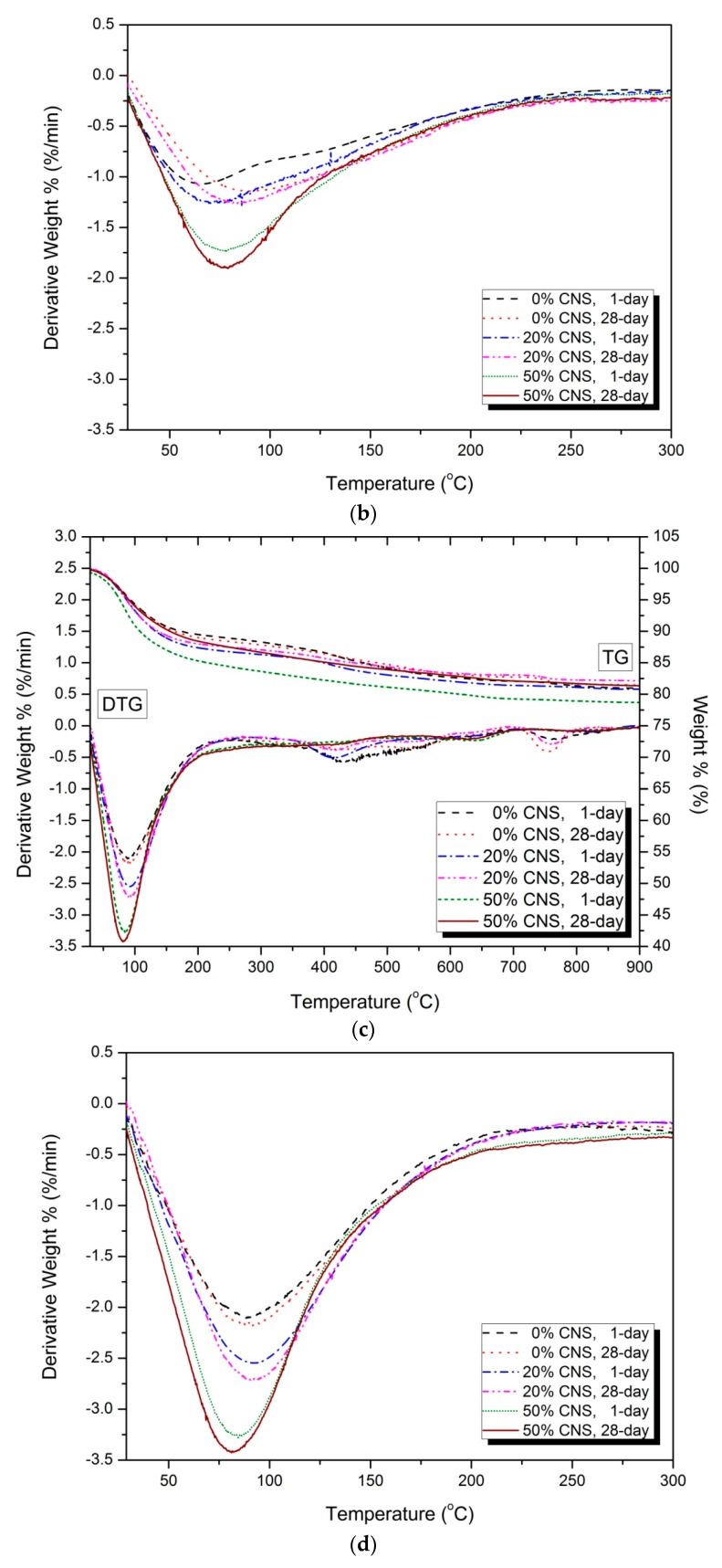
Thermal analysis (DTG): (**a**) 5% activator, (**b**) a magnification of less than 300 °C in (**a**), (**c**) 10% activator, (**d**) a magnification of less than 300 °C in (**c**).

Figure 8 shows the thermal analysis results for 1-day and 28-day hydration reactants. Figure 8a shows the DTG results for 1-day and 28-day hydration reactants of 0, 20, and 50% CNS samples of 5% activator. The weight loss rate seen at 50–200 °C is evaporation of water or C-S-H gel [50,51,52]. In addition, the weight loss rate observed at 130–200 °C is monosulfate [49]. The weight loss rate at 330–400 °C represents hydrotalcite-like phase [50,53], and the weight change between 665 and 800 °C means calcite [50,54,55]. Figure 8b shows an enlarged view of the temperature range of less than 300 °C to confirm the formation of AASC’s representative hydration reactants, C-S-H gel. The weight loss rate of the C-S-H gel at 28-day was slightly increased compared with that at 1-day, and the difference in the weight loss rates was not significant. This means that the CNS reactions mostly to the initial hydration stage of the AASC. As a result, the influence of CNS on the formation of hydration products after 1-day is estimated to be insignificant. Also, as the substitution ratio of CNS increases, the weight loss rate of C-S-H gel increases. Therefore, it shows the largest weight loss rate of 50% CNS.

Figure 8c shows the thermal analysis results of 10% activator. The results are similar to the thermal analysis results of the 5% activator samples. That is, as the substitution ratio of CNS increases, the weight loss rate of C-S-H gel in the range of 50–200 °C becomes larger. The comparison of the weight loss rate of C-S-H gel shown in Figure 8d shows that the formation amount of C-S-H gel of 28-day is larger than that of 1-day. This tendency is clear as the substitution rate of the CNS increases. Thermal analysis clearly shows the relationship between the formation of C-S-H gel and the rate of CNS replacement, which was not evident in the XRD analysis. This confirms that the substitution of the CNS affects the formation of C-S-H gel. In Figure 8, the difference in weight loss between 1-day and 28-day C-S-H gel was not significant in either the 5 and 10% activator samples. As a result, the effect of substitution of CNS on the formation of C-S-H gel is decreased after 1-day. Therefore, the XRD analysis described above shows a calcite-C-S-H gel peak with little change and tendency.

In the 5% and 10% activator, the weight loss in the 50–200 °C region increases as the substitution rate of the CNS increases. This is consistent with the tendency of the weight loss in the 50–200 °C region to increase with increasing contents of the nano-silica particles reported in previous studies [19,22,37]. However, a clear analysis of the thermal properties of AAC/gepolymer with nano-silica particles is still being attempted. In particular, the cause of the weight loss shown in the thermal analysis results in a temperature range lower than 200 °C is unclear. This is because the formation of hydration products by nano-silica particles and the effect of simple evaporation of water act simultaneously [19,22]. In Figure 5, the change in the C-S-H peak was negligible despite the increasing rate of substitution of CNS. Nevertheless, the pore size decreased and the compressive strength increased. This is due to the effect of promoting the formation of C-S-H gel by nano-silica and the filler effect. The increase in nano-silica contents requires a large amount of bound water [8,16,19,22]. Bound water by nano-silica particles evaporates at temperatures below 200 °C, causing weight loss. Therefore, weight loss in the temperature range of less than 200 °C shown in Figure 8 is due to the formation of C-S-H gel and increase of bound water content with increasing nano-silica particles. As a result, weight loss at a temperature of less than 200 °C shows a clear increase as the substitution rate of the CNS increases. However, the clear distinction and mechanism of these two effects still need further research.

The loss observed in the region of 350 to 550 °C in Figure 8d is estimated to be due to the hydrotalcite phase shown in the XRD analysis of Figure 4. In the XRD results of Figure 5, the 10% activator sample has the hydrotalcite peak that is relatively larger than the 5% activator. As a result, in Figure 8a of the 5% activator, the change in weight loss in the 350 to 550 °C region is negligible. In Figure 5c,d, the hydrotalcite peak decreased as the substitution ratio of CNS increased to 0, 20, and 50%. In addition, the peak intensity of hydratalcite at 28-day is lower than that at 1-day. In Figure 8c, 1-day samples of 0% CNS and 20% CNS clearly show weight loss in the 350 to 550 °C range. In addition, 28-day samples of 0% CNS and 20% CNS show small weight loss. However, 50% CNS samples showed little weight loss of hydrotalcite. Therefore, the tendency of hydrotalcite in XRD results was confirmed by thermal analysis (TG/DTG).

The weight loss in the region of 665–800 °C shown in Figure 8a,c shows complex characteristics. In Figure 8a, 1-day samples of 0% CNS and 20% CNS show the greatest weight loss. Greater weight loss than 28-day samples may be due to unexpected exposure to CO_2_ during sample storage, powder manufacture, and measurement. In Figure 8c, the weight loss rate decreases as the substitution ratio of CNS increases. The definite cause of this is difficult to confirm in the scope of this study. The reason is that unlike OPC, AAC has no portlandite [30], so the carbonate process is affected by various factors. To date, the carbonation of AAC has been associated with the pH reduction of the pore solution, the precipitation of Na-rich carbonates and the decalcification of Ca-rich phases (C-S-H gel) [56,57]. The CaCO_3_, which is formed as a result of carbonation, is classified into crystalline phases and an amorphous phase [57]. Therefore, CaCO_3_, which is distinguished by calcite, vaterite and aragonite, may be different from XRD and TG/DTG [57]. The weight loss in the region of 665–800 °C observed in Figure 8 is estimated to be the weight loss due to the carbonate, but the analysis of causes and trends is not clear in the scope of this study.

### 3.6. Microstructure

**Figure 9:**
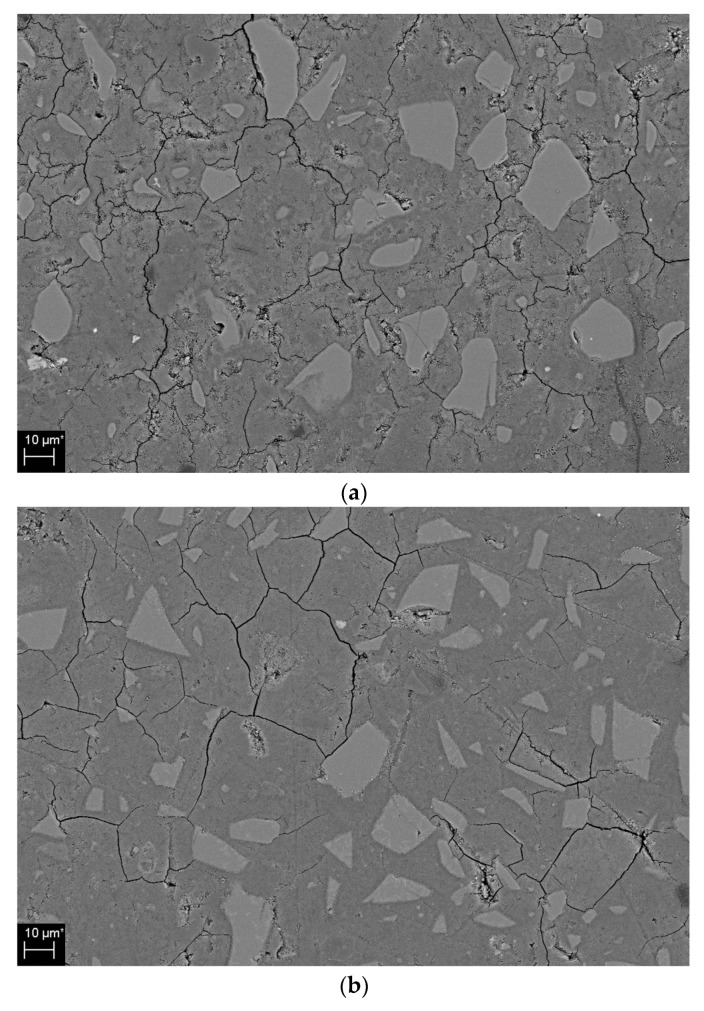

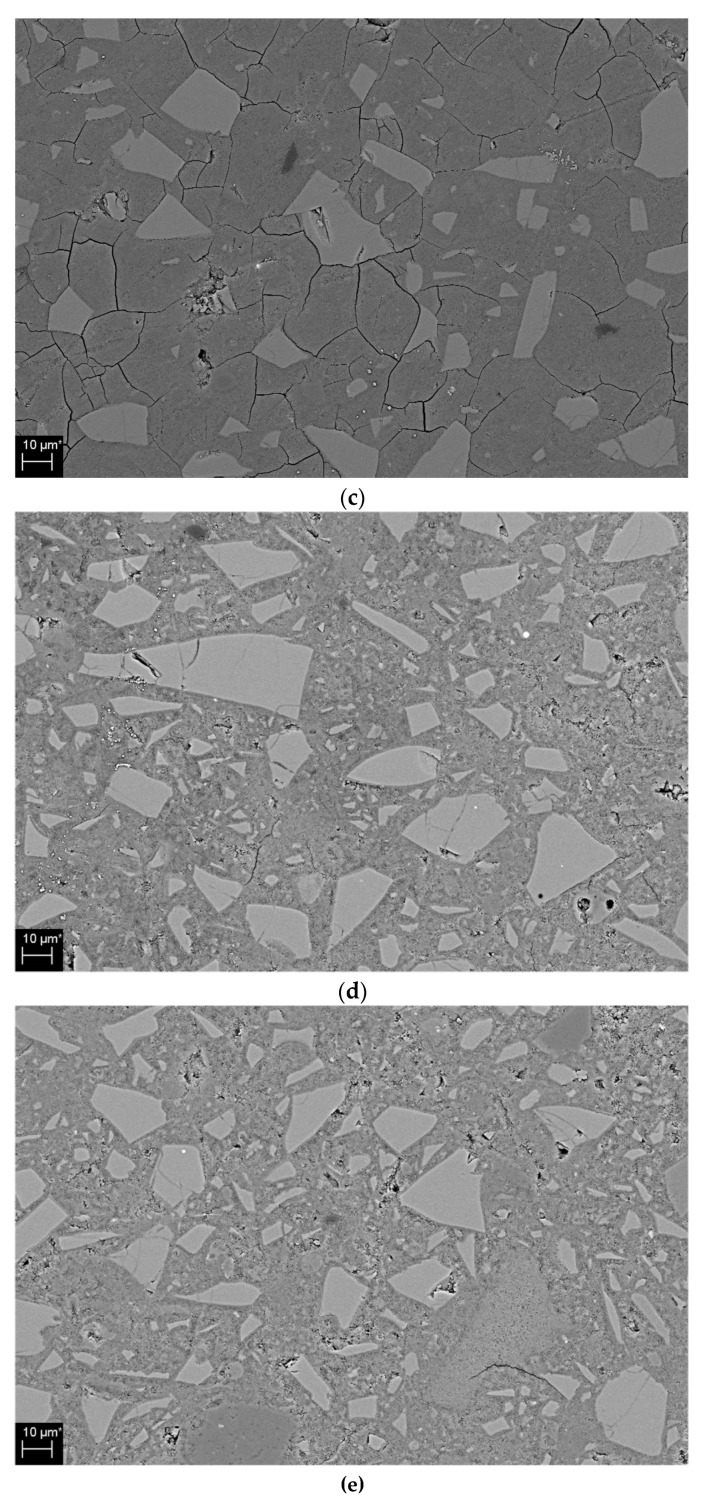

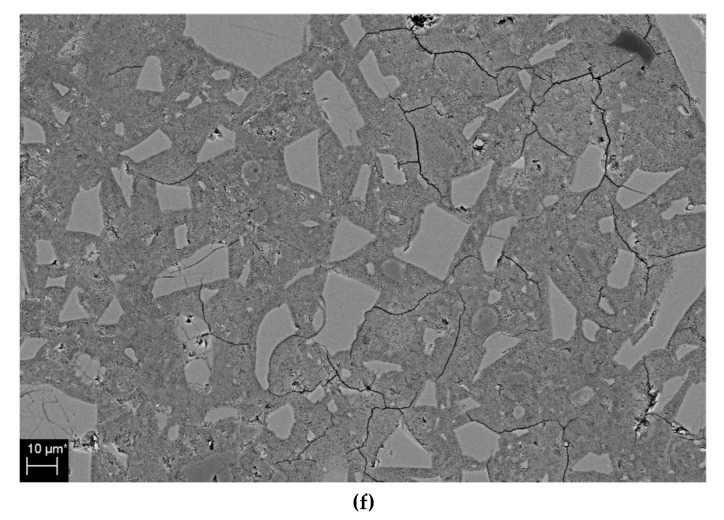
SEM/BSE image of hydration products: (**a**) 5% activator, 0% CNS, (**b**) 5% activator, 20% CNS, (**c**) 5% activator, 50% CNS, (**d**) 10% activator, 0% CNS, (**e**) 10% activator, 20% CNS, (**f**) 10% activator, 50% CNS.

Figure 9 shows SEM/BSE images of 0, 20, and 50% CNS in the 5 and 10% activator samples. The microcracks shown on the surface of the samples in Figure 9 occurred during the preprocessing and drying preparation for the observation of SEM/BSE samples. Compared to the 0% CNS shown in Figure 9a, the number and size of pores decreased in the 20 and 50% CNS shown in Figure 9b,c. In addition, as the substitution ratio of CNS increases, a more compact matrix is formed. Compared with Figure 9d, which is an SEM/BSE image for a 0% CNS of 10% activator, the formation of a dense matrix is confirmed at 20 and 50% CNS. Also, as the substitution ratio of CNS increases, the size and distribution of pores decrease. 

Pore reduction and dense matrix formation as the CNS replacement ratio increases are related to the improvement of compressive strength. Thus, the dense hydration reactants identified by SEM/BSE observations in Figure 8 support the results of the improved compressive strength shown in Figure 4 and Table 3.

Figure 9 is consistent with the results of a study of dense matrices through SEM in experiments on nano-particle substituted AAC/geopolymers [24,39,40,41,43].

## 4. Conclusions

The conclusions of characteristics of alkali-activated slag cement (AASC) mixed by mixing-water weight substitution method of colloidal nano-silica can be summarized as follows:

As the substitution rate of CNS increases, the liquidity and setting time decrease. However, the mixture was able to secure the minimum fluidity and working time required for compaction and molding. The 1-day compressive strength increase rate was the highest, the compressive strength increase rate decreased as the time increased, and the 28-day increase rate was the lowest. The highest 1-day compressive strength increase rate indicates that CNS has a great influence on the formation and densification of hydration products in the initial hydration stage. This is in agreement with previous studies that colloidal nano-silica shows faster reactivity at the initial hydration stage than powdered nano-silica. CNS has the greatest effect on the change of pore structure. Increased CNS replacement rates decrease the volume of medium capillary pores (50–10 nm) drastically and increase the volume of gel pores (< 10 nm). As a result, the CNS has the effect of reducing the size and volume of pores and making the matrix compact. In addition, XRD, TG/DTG and SEM analysis supported the formation of dense matrix by substitution of CNS.

The newly applied substitution method in this study can explain the changes in mechanical properties and microstructure in the properties of AASC due to the following reasons. Replacing a portion of the mixing water with the CNS results in uniform dispersion of the nano-silica particles, resulting in improved nucleation and filler effects. Also, substitution of CNS with higher density than mixing water has the effect of decreasing w/b ratio. The dispersion of homogeneous nano-silica particles and the reduction of w/b ratio were important factors affecting the properties of AASC. Therefore, the new mixing method could be applied to the mix design of AASC nano-silica. Based on the experimental results, it is expected that the application range can be extended to other kinds of colloidal nano-particles or mixtures with higher substitution ratio.

## Figures and Tables

**Table 1:** Properties of slag.

	Chemical Components (%)							Density (g/cm^3^)	Fineness (m^2^/kg)	LOI (%)
	SiO_2_	Al_2_O	Fe_2_O	MgO	CaO	K_2_O	SO_3_			
slag	21.32	5.61	3.12	3.94	61.72	0.79	2.51	2.84	420	1.86

**Table 2:** Summary of w/b ratios, mixing ratio of CNS and mixture proportions.

w/b Considering Water Including CNS	CNS Replacement Ratios		Ratio of SiO_2_ in CNS to Binder Weight (%)	Slag (g)	Mixing Water (g)	CNS (g)
	CNS to Mixing Water Ratio (%)	CNS to Binder Ratio (%)				
0.500	0	0	0	1000	500	0
0.485	10	5	1.5	1000	450	50
0.470	20	10	3.0	1000	400	100
0.450	30	15	4.5	1000	350	150
0.440	40	20	6.0	1000	300	200
0.425	50	25	7.5	1000	250	250

**Table 3:** Relative strength change rate compared to without CNS sample.

Contents of CNS (%)	5% Activator				10% Activator			
	1-day	3-day	7-day	28-day	1-day	3-day	7-day	28-day
10	20.10	18.69	23.41	19.42	110.54	88.47	47.95	37.10
20	78.31	64.65	58.22	52.01	246.14	199.18	114.26	85.23
30	157.45	140.08	106.07	84.78	285.25	226.35	143.17	113.44
40	233.73	255.44	186.63	156.24	334.97	305.30	205.35	172.43
50	277.25	296.44	230.43	177.52	375.07	346.11	238.04	188.00

**Table 4:** Pore size distribution of CNS contents.

Activator (%)	CNS Content (%)	Large Capillary Pores (10,000‒50 nm) (%)		Medium Capillary Pores (50‒10 nm) (%)		Gel Pores (<10 nm) (%)	
		1-day	28-day	1-day	28-day	1-day	28-day
5	0	3.49	3.94	57.25	56.34	39.26	39.72
	20	3.34	3.59	46.66	44.66	50.00	51.75
	50	7.56	2.17	6.98	5.02	85.46	92.80
10	0	3.06	1.07	32.06	31.22	64.88	67.71
	20	8.53	2.50	6.43	5.31	85.05	92.71
	50	1.65	1.16	0.00	0.00	98.35	98.84
